# Contraceptive Method Mix and HIV Risk Behaviors Among Kenyan Adolescent Girls and Young Women Seeking Family Planning Services: Implications for Integrating HIV Prevention

**DOI:** 10.3389/frph.2021.667413

**Published:** 2021-07-21

**Authors:** Edward Nyaboe, Anna Larsen, Joseph Sila, John Kinuthia, George Owiti, Felix Abuna, Pamela Kohler, Grace John-Stewart, Jillian Pintye

**Affiliations:** ^1^Kenya Medical Research Institute, Nairobi, Kenya; ^2^Department of Epidemiology, University of Washington, Seattle, WA, United States; ^3^Department of Research and Programs, Kenyatta National Hospital, Nairobi, Kenya; ^4^Department of Global Health, University of Washington, Seattle, WA, United States; ^5^Department of Child, Family, and Population Health, University of Washington, Seattle, WA, United States; ^6^Department of Medicine, University of Washington, Seattle, WA, United States; ^7^Department of Pediatrics, University of Washington, Seattle, WA, United States; ^8^Department of Biobehavioral Nursing and Health Informatics, University of Washington, Seattle, WA, United States

**Keywords:** contraceptive use, LARC, HIV prevention, adolescents, Africa

## Abstract

**Background:** Understanding HIV risk behaviors among adolescent girls and young women (AGYW) seeking contraception could help inform integrating HIV prevention services within family planning (FP) clinics.

**Methods:** From 10/2018 to 04/2019, we conducted a survey at 4 FP clinics in Kisumu, Kenya to evaluate risk behaviors among AGYW without HIV infection seeking contraception. All AGYW aged 15–24 were invited to participate following receipt of FP services. Adolescent girls and young women initiating or refilling contraception were included in this analysis. Long-acting reversible contraceptives (LARC) included intrauterine devices, implants, or injectables. Non-LARC methods included oral contraceptive pills (OCP) or condoms. We used an empiric risk score to assess HIV risk behaviors; HIV risk scores of ≥5 (corresponding to 5–15% HIV incidence) defined “high” HIV risk.

**Results:** Overall, 555 AGYW seeking FP were included. Median age was 22 years [interquartile range (IQR) 20–23], median completed education was 12 years (IQR 10–12); 23% of AGYW had HIV risk scores of ≥5. The most frequent form of contraception was injectables (43%), followed by implants (39%). After adjustment for education, prior pregnancy, and marital status, LARC users more frequently engaged in transactional sex than non-LARC users [6 vs. 0%, adjusted prevalence ratio (PR) = 1.17, 95% CI 1.09–1.29, *p* < 0.001]; LARC use was not associated with HIV risk scores ≥5. Among LARC users, AGYW using injectables more frequently had condomless sex compared to AGYW using other LARC methods (85 vs. 75%, adjusted PR = 1.52, 95% CI 1.09–2.10, *p* = 0.012); injectable use was not associated with HIV risk scores ≥5.

**Conclusions:** Adolescent girls and young women seeking contraception frequently had high HIV risk, emphasizing the importance of integrating HIV prevention within FP. Multipurpose technologies for contraception and HIV prevention could particularly benefit AGYW.

## Introduction

Long-acting reversible contraceptives (LARCs) have the potential to reduce unintended pregnancy and associated morbidity and mortality, particularly in high HIV prevalence settings of sub-Saharan Africa where 47% of women have an unmet need for modern contraception (1, 2). Following goals set out by the Family Planning 2020 (FP2020) initiative, LARC access is expanding in sub-Saharan Africa, with younger and less educated women reached through demand generation approaches and service delivery mechanisms (3, 4). Results from the recent ECHO (Evidence for Contraceptive Options in HIV Outcomes) randomized trial among women recruited through family planning (FP) clinics in eSwatini, Kenya, South Africa, and Zambia provide strong evidence that HIV acquisition risk does not substantially differ between LARC methods commonly used in African settings (5). However, ECHO found an alarmingly high HIV incidence rate (4.3%) among adolescent girls and young women (AGYW) despite an individualized HIV prevention package provided to all participants and country-wide HIV treatment and prevention programs (5). These findings highlight a gap in integration of HIV prevention services for AGYW into routine FP care.

Behavioral risks for HIV acquisition may differ among AGYW who self-select certain FP methods over others in real-world settings. Understanding behavioral profiles among AGYW seeking contraception could help inform integration of tailored HIV prevention counseling and interventions within FP clinics. We evaluated the contraceptive method mix and HIV behavioral risk factors among AGYW seeking FP services at routine clinics in Kisumu County, Kenya.

## Methods

### Study Setting and Design

The PrIYA Program, a collaboration with the Department of Health and Sanitation, Kisumu County, and the National AIDS and STI Control Programme (NASCOP), was a 2-year implementation project which integrated delivery of PrEP into routine maternal child health and FP systems (6–8). The program aimed to reach AGYW at high risk for HIV acquisition and was implemented from June 2017 to October 2018 in 16 facilities in Kisumu County, Kenya, which has an adult HIV prevalence of 19.9%. (9–11). We conducted a survey at a subset of former PrIYA sites to evaluate behavioral characteristics and HIV risk factors among AGYW in FP clinics (12, 13). Four public-sector facilities were selected based on having the highest monthly enrollment of new FP clients.

### Study Population

All HIV-negative women at the four facilities were approached after receipt of routine FP services, including HIV testing, from October 2018 to June 2019. Those between 15 and 24 years and who received FP services at the facility, including confirmation of HIV-negative status via routine HIV testing, were eligible for enrollment. All eligible women interested in participating were enrolled upon provision of written informed consent. Adolescent girls and young women were included in the current analysis if they initiated or refilled an FP method, including injectables, implants, IUDs, oral contraceptive pills (OCP), or condoms. We excluded AGYW who were removing a contraceptive method or seeking other non-contraceptive services (e.g., cervical cancer screening) at the FP clinics.

### Data Collection

Trained study nurses administered surveys in Kiswahili, Dholuo, or English using tablets. Surveys were field-tested and included questions about demographics, partnership characteristics, sexual and reproductive behaviors, perceived HIV risk, and HIV risk behaviors. Long-acting reversible contraceptive was defined as IUDs, implants, or injectables. Non-LARC methods included OCP or condoms. Contraceptive type was mutually exclusive and defined as the primary method used for contraception (e.g., no dual methods).

### Behavioral HIV Risk Assessment

We evaluated participants for HIV behavioral risk factors using a standardized risk assessment tool used by the Kenya Ministry of Health to screen for PrEP which includes the following behavioral characteristics: partner HIV status, condomless sex, engagement in transactional sex, experiencing intimate partner violence, and being forced to have sex in the last 6 months (14). We used an empiric risk score to further assess HIV risk behaviors which was validated to predict risk of HIV acquisition among young women in sub-Saharan African settings (15). Characteristics included in the risk score were age <25 years old (risk score of 2), not living with a spouse/partner (1), any alcohol use within the past 30 days (1), receiving financial support from a partner (1), and having a partner with other sexual partners (2) or not knowing if a partner has other sexual partners (1, 15). “High” HIV risk is defined by an HIV risk score of ≥5 (corresponding to 5–15% HIV incidence in cohorts of African women) (15). Risk scores of ≤4 correspond to HIV incidence of 0–5% and are considered “low” HIV risk. We also assessed self-perceived risk for HIV acquisition on a four-point Likert scale by asking participants “*What is your gut feeling about how likely you are to get infected with HIV?*,” with possible responses of very likely, somewhat likely, very unlikely, or extremely unlikely (16).

### Statistical Analysis

We used descriptive statistics to determine the frequency of demographic characteristics, pregnancy history and FP use, HIV risk perception, and HIV risk behaviors (15). We used Poisson regression models, clustering by facility, to calculate prevalence ratios (PRs) for HIV risk factors by LARC use status. Potential correlates of LARC use identified in univariable models were adjusted for years completed education, having at least one prior pregnancy, and marital status in multivariable models; adjustment variables were determined *a priori* because of their known association with LARC use based on prior studies. We used similar models to calculate PRs for HIV risk factors by injectable use status among LARC users. Analyses were performed in STATA 15.0.

### Considerations for Human Subjects

The Kenyatta National Hospital-University of Nairobi Ethics Research Committee and University of Washington Human Subjects Review Committee reviewed and approved the study protocol, informed consent forms, and data collection tools. We also obtained approval by the Kisumu County Department of Health and health administrators within the health facilities involved.

## Results

**Figure 1:**
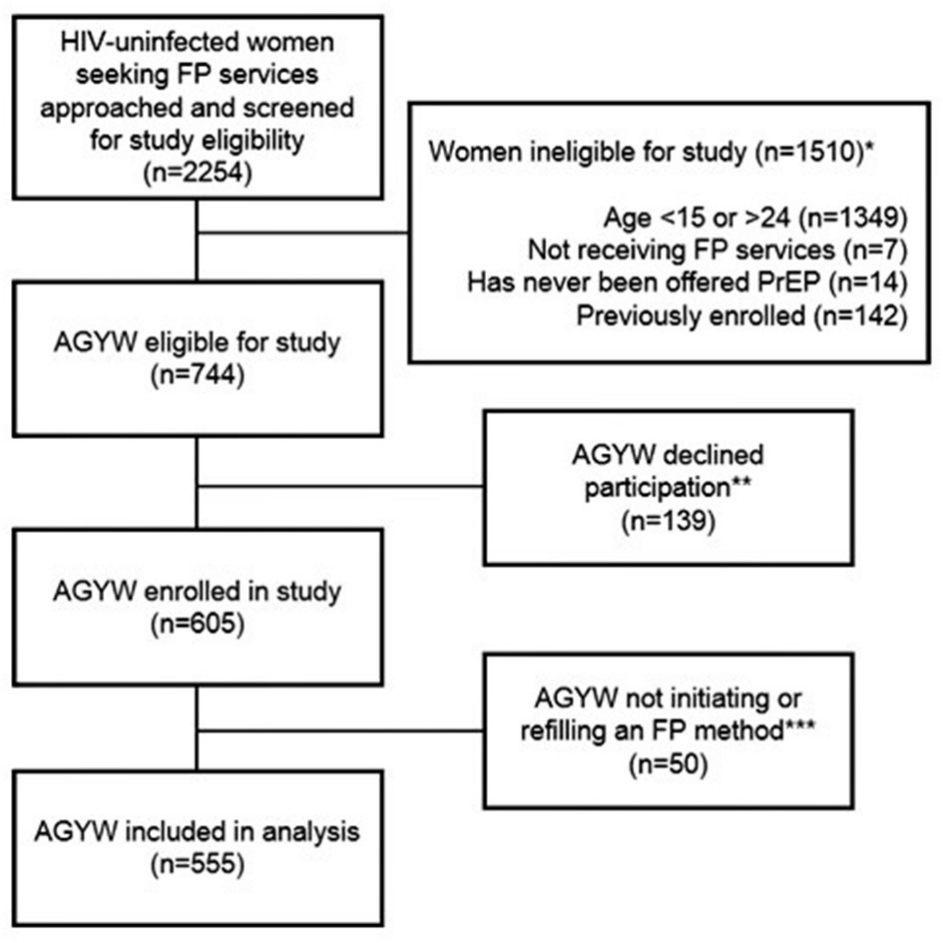
Flow chart of participant inclusion in the present analysis among HIV-uninfected AGYW seeking family planning services in Western Kenya. AGYW, adolescent girls and young women. ^*^Categories describing reasons for ineligibility are not mutually exclusive. ^**^Reasons for declination were not captured systematically. Anecdotally, the most common reason for declining was lack of time. Other common reasons included infant crying/fussing and male partner refusal. ^***^Fifty women were not initiating or refilling a family planning method, thus were excluded from the present analysis.

Overall, 555 AGYW seeking FP services (initiating or refilling an FP method) completed the survey, and were included in this analysis (Figure 1). Median age was 22 years [interquartile range (IQR) 20–23], median completed education was 12 years (IQR 10–12), 24% of women were currently in school, and 59% were married. The majority 464 (84%) of AGYW had a current primary partner, of whom 87% reported their partner was HIV-negative and 12% reported not knowing their partner's HIV status; 4 (1%) AGYW reported having a partner known to be living with HIV. Approximately one-fourth (23%) of AGYW had HIV risk scores ≥5.

The most frequent form of contraception was injectables (43%), followed by implants (39%), pills (12%), intrauterine devices (3%), and condoms alone (3%). Long-acting reversible contraceptive use was associated with years of completed education and having a prior pregnancy (Table 1). There were no differences in frequency of HIV risk scores ≥5 between AGYW using LARC compared to those using non-LARC methods. Adolescent girls and young women who used LARC more frequently reported engaging in transactional sex in the last 6 months compared to non-LARC methods users (6 vs. 0%, adjusted PR = 1.17, 95% CI 1.09–1.29, *p* < 0.001). There were no differences in other behavioral risk factors for HIV between LARC and non-LARC users.

**Table 1:** Demographic characteristics and HIV behavioral risk factors among LARC and non-LARC contraceptive users (*n* = 555)^a^.

**Characteristic**	**Overall** **(*n* = 555)**	**Contraceptive type**		**Univariate Poisson regression**		**Multivariate Poisson regression**	
		**LARC** **(*n* = 475)**	**Non-LARC** **(*n* = 80)**	**Unadjusted PR** **(95% CI)**	***p*-value**	**Adjusted PR** **(95% CI)**	***p*-value** ^**b**^
**DEMOGRAPHIC CHARACTERISTICS**							
Age ≥22 years	285 (51.4%)	253 (53.3%)	32 (40.0%)	1.08 (0.99–1.17)	0.066		
Completed education ≤12 years	423 (76.2%)	355 (74.7%)	68 (85.0%)	1.08 (1.03–1.14)	0.002	1.08 (1.01–1.16)	0.020
Currently in school	121 (21.9%)	98 (20.7%)	23 (28.7%)	0.93 (0.85–1.02)	0.136		
Regularly employed	80 (14.5%)	73 (15.5%)	7 (8.8%)	1.08 (1.06–1.10)	<0.001	1.00 (0.93–1.07)	0.962
Currently has primary partner	464 (83.6%)	394 (82.9%)	70 (87.5%)	0.96 (0.85–1.08)	0.446		
At least one prior pregnancy	446 (80.4%)	402 (84.6%)	44 (55.0%)	1.35 (1.23–1.47)	<0.001	1.34 (1.20–1.48)	<0.001
**BEHAVIORAL HIV RISK FACTORS**							
Total lifetime sexual partners (≥4)	83 (15.0%)	74 (15.6%)	9 (11.3%)	1.05 (1.00–1.10)	0.044	0.99 (0.92–1.07)	0.885
Partner HIV status unknown or positive	62 (13.4%)	50 (12.8%)	12 (17.1%)	0.94 (0.87–1.01)	0.128		
Condomless sex (last 6 months)	437 (78.7%)	380 (80.0%)	57 (71.3%)	1.08 (1.02–1.15)	0.012	1.02 (0.98–1.06)	0.422
Transactional sex (last 6 months)	28 (5.0%)	28 (5.9%)	0 (0.0%)	1.18 (1.04–1.34)	0.009	1.17 (1.09–1.24)	<0.001
Forced sex (last 6 months)	38 (6.8%)	35 (7.4%)	3 (3.8%)	1.08 (0.94–1.25)	0.275		
Intimate partner violence^c^	12 (2.6%)	11 (2.8%)	1 (1.4%)	1.08 (1.01–1.16)	0.031	1.04 (0.93–1.15)	0.481
High self-perceived HIV risk^d^	76 (13.7%)	65 (13.7%)	11 (13.8%)	1.00 (0.93–1.07)	0.990		
**EMPIRIC HIV RISK SCORE FACTORS**							
Unmarried/Not living with partner	228 (41.1%)	180 (37.9%)	48 (60.0%)	0.94 (0.88–0.99)	0.026	0.99 (1.20–1.48)	0.573
Alcohol use (past 30 days)	75 (13.5%)	65 (13.7%)	10 (12.5%)	1.01 (0.95–1.08)	0.655		
No financial support from partner	12 (2.2%)	10 (2.1%)	2 (2.5%)	0.97 (0.66–1.43)	0.890		
Primary partner has other partners	203 (36.6%)	174 (36.6%)	29 (36.3%)	1.00 (0.98–1.03)	0.929		
High HIV risk (risk score: ≥5)^e^	125 (22.5%)	99 (20.8%)	26 (32.5%)	0.91 (0.86–0.86)	0.001	0.98 (0.93–1.02)	0.326

*LARC, long-acting reversible contraception*.

a *Other LARC methods include implants and intrauterine devices*.

b *Prevalence ratios adjusted for years completed education, having at least one prior pregnancy, and marital status*.

c *Intimate partner violence defined as Hurt-Insult-Threaten-Scream (HiTS) score ≥10 (32)*.

d *High self-perceived HIV risk, Somewhat/very likely to acquire HIV; Low self-perceived HIV risk, Extremely/very unlikely to acquire HIV*.

e *VOICE risk scoring (16): Age < 25 = 1 (all participants in this analysis are <25, thus we have excluded age from the table but included the age score in the risk score calculation), Married = 2, any alcohol = 1, partner provides financial support = 1, partner has other partners: yes = 2, do not know = 2*.

Among AGYW using LARC (*n* = 460), injectable users were less frequently to report being currently in school and a prior pregnancy (Table 2). There were no differences in frequency of HIV risk scores ≥5 between AGYW using injectables compared to those using other LARC methods. Among other individual HIV risk behaviors, AGYW using injectables more frequently had condomless sex in the last 6 months compared to AGYW using other LARC methods (85 vs. 75%, adjusted PR = 1.52, 95% CI 1.09–2.10, *p* = 0.012), yet less frequently had ≥4 lifetime sexual partners (12 vs. 20%, adjusted PR = 0.75, 95% CI 0.64–0.88, *p* < 0.001). There were no other differences in HIV risk behaviors between AGYW using injectables compared to those using other LARC methods.

**Table 2:** Demographic characteristics and HIV behavioral risk factors among injectable and other LARC users (*n* = 475)^a^.

**Characteristic**	**Overall** **(*n* = 475)**	**Contraceptive type**		**Univariate Poisson regression**		**Multivariate Poisson regression**	
		**Injectable** **(*n* = 240)**	**Other LARC** **(*n* = 235)**	**Unadjusted PR** **(95% CI)**	***p*-value**	**Adjusted PR** **(95% CI)**	***p*-value** ^**b**^
**DEMOGRAPHIC CHARACTERISTICS**							
Age ≥22 years	253 (53.3%)	124 (52.8%)	129 (53.8%)	1.01 (0.89–1.16)	0.766		
Completed education ≤12 years	355 (74.7%)	181 (75.4%)	174 (74.0%)	0.96 (0.71–1.31)	0.814		
Currently in school	98 (20.7%)	43 (18.1%)	55 (23.4%)	0.84 (0.79–0.90)	<0.001	0.72 (0.64–0.80)	<0.001
Regularly employed	73 (15.5%)	41 (17.2%)	32 (13.7%)	1.14 (1.05–1.24)	0.002	1.21 (0.97–1.50)	0.095
Currently has primary partner	394 (82.9%)	200 (83.3%)	194 (82.6%)	1.03 (0.79–1.33)	0.835		
At least one prior pregnancy	402 (84.6%)	193 (80.4%)	209 (88.9%)	0.75 (0.67–0.83)	<0.001	0.63 (0.53–0.74)	<0.001
**BEHAVIORAL HIV RISK FACTORS**							
Total lifetime sexual partners (≥4)	74 (15.6%)	28 (11.7%)	46 (19.6%)	0.72 (0.57–0.89)	0.003	0.75 (0.64–0.88)	<0.001
Partner HIV status unknown or positive	50 (12.8%)	42 (19.0%)	32 (14.6%)	1.25 (0.93–1.69)	0.133		
Condomless sex (last 6 months)	380 (80.0%)	205 (85.4%)	175 (74.5%)	1.46 (1.06–2.03)	0.022	1.52 (1.09–2.10)	0.012
Transactional sex (last 6 months)	28 (5.9%)	12 (5.0%)	16 (6.8%)	0.84 (0.73–0.96)	0.013	0.93 (0.74–1.16)	0.523
Forced sex (last 6 months)	35 (7.4%)	17 (7.1%)	18 (7.7%)	0.96 (0.85–1.08)	0.475		
Intimate partner violence^c^	11 (2.8%)	5 (2.5%)	6 (3.1%)	0.89 (0.64–1.24)	0.497		
High self-perceived HIV risk^d^	65 (13.7%)	33 (13.8%)	32 (13.6%)	1.01 (0.82–1.24)	0.940		
**EMPIRIC HIV RISK SCORE FACTORS**							
Unmarried/not living with partner	180 (37.9%)	89 (37.1%)	91 (38.7%)	0.98 (0.92–1.05)	0.630		
Alcohol use (past 30 days)	65 (13.7%)	36 (15.0%)	29 (12.3%)	1.11 (0.87–1.42)	0.384		
No financial support from partner	10 (2.1%)	235 (97.9%)	230 (97.9%)	0.99 (0.61–1.61)	0.966		
Primary partner has other partners	174 (36.6%)	88 (36.7%)	86 (36.6%)	1.00 (0.95–1.06)	0.979		
High HIV risk (risk score: ≥5)^e^	99 (20.8%)	51 (21.3%)	48 (20.4%)	1.02 (0.93–1.12)	0.603		

*LARC, long-acting reversible contraception*.

a *Other LARC methods include implants and intrauterine devices*.

b *Prevalence ratios adjusted for years completed education, having at least one prior pregnancy, and marital status*.

c *Intimate partner violence defined as Hurt-Insult-Threaten-Scream (HiTS) score ≥10 (34)*.

d *High self-perceived HIV risk, Somewhat/very likely to acquire HIV; Low self-perceived HIV risk, Extremely/very unlikely to acquire HIV*.

e *VOICE risk scoring (16): Age <25 = 1 (all participants in this analysis are <25, thus we have excluded age from the table but included the age score in the risk score calculation), Married = 2, any alcohol = 1, partner provides financial support = 1, partner has other partners: yes = 2, do not know = 2*.

Overall, 14% of AGYW reported that they felt acquiring HIV in the next year was very likely. AGYW with partners of unknown HIV status or who were known to be living with HIV were more likely to report high self-perceived HIV risk than AGYW with HIV-negative partners (42 vs. 8%, PR = 1.31, 95% CI 1.24–1.39, *p* < 0.001). There were no differences in high self-perceived HIV risk among AGYW with risk scores ≥5 compared to those with scores <5 (18 vs. 12%, PR = 1.05, 95% 98–1.13, *p* = 0.166). There were also no appreciable differences in HIV risk perception across contraceptive methods (data not shown).

## Discussion

In this survey among Kenyan AGYW within routine FP settings, LARC use was frequent with >80% of AGYW using either injectables or implants. Nearly one-quarter of AGYW had HIV risk scores ≥5, indicating high behavioral risk for HIV acquisition (15), though only 14% of AGYW felt acquiring HIV in the next year was very likely. Our results add to recent data underscoring that AGYW seeking FP services frequently have behavioral risks for HIV acquisition and that differences between AGYW who self-select certain FP methods are important considerations for HIV prevention interventions. Our findings support the need to integrate HIV prevention services within FP with tailored counseling for AGYW. Given the high frequency of LARC methods observed in our study population, long-acting PrEP agents and multipurpose technologies may be particularly attractive in this setting (17).

In our study, report of condomless sex in the last 6 months was high (80%), similar to the ECHO trial in which 73% of participants recruited from FP clinics reported condomless sex in the last 3 months (5). We found that AGYW using injectables more frequently reported condomless sex than AGYW using other LARC. Prior to the ECHO trial, observational studies evaluating the causal relationship between DMPA and HIV risk were prone to concerns about confounding factors, such as underreported condomless sex (18). Studies evaluating biomarkers of condomless sex among women in Zimbabwe demonstrated that misreporting of condom use does not differ between injectable, OCP, or non-hormonal contraception users, though implants users were not evaluated (19). Our results suggest that AGYW who self-select injectable contraception may be more likely to have condomless sex and subsequently higher HIV risk in real-world settings. Adolescent LARC users may no longer perceive a need for condoms if the likelihood of pregnancy is minimal, even if they have other HIV risk behaviors (20). As uptake of LARC increases among AGYW, there is an urgent need to incorporate HIV prevention services like PrEP within FP settings (7). Multipurpose prevention technologies (MPTs) in the pipeline for prevention of HIV and unintended pregnancy in one formulation could be particularly useful for AGYW who may benefit from HIV prevention tools and for whom condoms are not preferred (21).

Similar to previous studies, we found that only a small proportion of AGYW in FP settings self-perceive high HIV risk, despite frequently reporting HIV risk behaviors (7, 22). Family planning providers are well-positioned to counsel AGYW on HIV risk behaviors and to ensure AGYW are aware of and offered comprehensive HIV prevention options (23). In the PrIYA Program (7, 8, 24), 16% of AGYW with HIV risk factors accepted PrEP (7, 8) and low perceived HIV risk was the primary reason for declining PrEP. Among the current study population, we previously reported PrEP uptake was 4% overall under programmatic conditions and 78% of AGYW with high behavioral HIV risk declined PrEP due to low perceived risk of HIV (13). To date, studies evaluating integrated delivery of FP with HIV prevention services primarily focus on provision of biomedical interventions such as PrEP and HIV testing (7, 25, 26). Interventions promoting confidentiality, supportive provider interaction, specialized provider training, and the removal of logistic barriers have positive effects on reproductive health outcomes among AGYW (27), though more rigorous research is needed. One ongoing study tests a standardized patient actor-based provider training to improve PrEP counseling for AGYW in Kenya and case scenarios include navigation of HIV risk assessment (28). Our findings support that counseling on HIV prevention within FP settings should consider how risk perception influences uptake of HIV prevention services among AGYW. More research is needed on that moves beyond provision of HIV prevention interventions to address factors influencing uptake within FP settings such as low risk perception.

Our study has limitations. We ascertained the primary FP method being initiated or refilled during the participant's clinic visit and did not assess dual use of condoms with other methods. However, condomless sex was very frequently reported in our study. Frequency of some HIV risk behaviors was rare (e.g., transactional or forced sex) and therefore our statistical power was limited to detect some associations. Our data are limited to AGYW seeking contraception at public sector facilities and may not be representative of AGYW who seek contraception elsewhere (e.g., retail pharmacies).

In conclusion, our results support that approaches currently in development to concurrently prevent HIV and unintended pregnancy may be particularly beneficial for AGYW, especially those who prefer injectable contraception and report condomless sex. Counseling on behavioral risks and HIV prevention tailored to AGYW could be useful within FP settings. More implementation research is needed on integrating HIV prevention services into FP to address other factors influencing uptake.

## Data Availability Statement

The raw data supporting the conclusions of this article will be made available by the authors, without undue reservation.

## Ethics Statement

The studies involving human participants were reviewed and approved by The Kenyatta National Hospital-University of Nairobi Ethics Research Committee and University of Washington Human Subjects Review Committee reviewed and approved the study protocol, informed consent forms, and data collection tools. We also obtained approval by the Kisumu County Department of Health and health administrators within the health facilities involved. Written informed consent from the participants' legal guardian/next of kin was not required to participate in this study in accordance with the national legislation and the institutional requirements.

## Author Contributions

GJ-S and JP designed the study. EN, JS, and AL analyzed the data. EN, JP, and AL drafted the manuscript. JK, GO, FA, PK, and GJ-S contributed to the interpretation of the results and critically revising the manuscript for important intellectual content, and all authors approved the manuscript for publication. All authors contributed to the article and approved the submitted version.

## Conflict of Interest

The authors declare that the research was conducted in the absence of any commercial or financial relationships that could be construed as a potential conflict of interest.
